# Treatment of intracerebral hemorrhage: From specific interventions to bundles of care

**DOI:** 10.1177/1747493020964663

**Published:** 2020-10-15

**Authors:** Adrian R Parry-Jones, Tom J Moullaali, Wendy C Ziai

**Affiliations:** 1 Manchester Centre for Clinical Neurosciences, Salford Royal NHS Foundation Trust, Salford, UK; 2 Division of Cardiovascular Sciences, The University of Manchester, Manchester, UK; 3 Centre for Clinical Brain Sciences, University of Edinburgh, Scotland, UK; 4 George Institute for Global Health, Sydney, Australia; 5 Division of Neurosciences Critical Care, Department of Neurology, Anesthesiology and Critical Care Medicine, Johns Hopkins University School of Medicine, Baltimore, MD, USA

**Keywords:** Intracerebral hemorrhage, antiplatelet drugs, anticoagulants, blood pressure, neurosurgery, critical care, care bundles

## Abstract

Intracerebral hemorrhage (ICH) represents a major, global, unmet health need with few treatments. A significant minority of ICH patients present taking an anticoagulant; both vitamin-K antagonists and increasingly direct oral anticoagulants. Anticoagulants are associated with an increased risk of hematoma expansion, and rapid reversal reduces this risk and may improve outcome. Vitamin-K antagonists are reversed with prothrombin complex concentrate, dabigatran with idarucizumab, and anti-Xa agents with PCC or andexanet alfa, where available. Blood pressure lowering may reduce hematoma growth and improve clinical outcomes and careful (avoiding reductions ≥60 mm Hg within 1 h), targeted (as low as 120–130 mm Hg), and sustained (minimizing variability) treatment during the first 24 h may be optimal for achieving better functional outcomes in mild-to-moderate severity acute ICH. Surgery for ICH may include hematoma evacuation and external ventricular drainage to treat hydrocephalus. No large, well-conducted phase III trial of surgery in ICH has so far shown overall benefit, but meta-analyses report an increased likelihood of good functional outcome and lower risk of death with surgery, compared to medical treatment only. Expert supportive care on a stroke unit or critical care unit improves outcomes. Early prognostication is difficult, and early do-not-resuscitate orders or withdrawal of active care should be used judiciously in the first 24–48 h of care. Implementation of acute ICH care can be challenging, and using a care bundle approach, with regular monitoring of data and improvement of care processes can ensure consistent and optimal care for all patients.

## Introduction

Intracerebral hemorrhage (ICH) represents a major cause of morbidity and mortality on a global scale. Of the 12 million incident strokes worldwide in 2017, over a quarter were due to ICH and because of the relatively high case fatality of ICH, hemorrhagic stroke accounts for just over half of all global stroke deaths.^1,2^ Considerable regional differences exist in incidence, prevalence, deaths, and disability, with much of the global burden of ICH residing in Asia, Africa, and Eastern Europe.^1^

Hypertension is the key modifiable risk factor for ICH, accounting for over half of the population attributable risk.^3^ Whilst improvements in the management of this key risk factor would be expected to have reduced ICH incidence in high income countries, ICH incidence has remained largely unchanged over the last 30 years,^1^ perhaps due to a rising incidence of cerebral amyloid angiopathy-related ICH associated with antithrombotic use in the elderly.^4^

Despite the considerable and ongoing burden of ICH, there remain few effective therapies when compared to ischemic stroke. However, evidence-based treatments when combined may have a considerable impact on outcome, and consistent and effective implementation can be supported by a “care bundle” approach, such as the recently described “ABC” care bundle for ICH.^5^ Here, we review the key interventions recommended in the first 72 h of ICH care and consider how these interventions can be effectively implemented. To aid clarity for the practicing clinician, we present these interventions in the order in which we believe they should be considered and delivered in the majority of ICH patients, focusing initially on reversal of antithrombotic drugs, followed by acute management of blood pressure (BP), then consideration for neurosurgical intervention, and finally deciding the appropriate level of ongoing supportive care for each patient.

## Antithrombotic medications

Around a third of ICH patients are taking anticoagulants or antiplatelet drugs at ICH onset and management of this issue has been further complicated by the introduction of the direct oral anticoagulants (DOACs) in addition to vitamin-K antagonists (VKAs). VKA-ICH patients tend to be older and have a larger hematoma at baseline, a three-fold higher odds of hematoma expansion and a higher mortality when compared to those not taking anticoagulants.^6^ DOAC-ICH appears to be associated with lower baseline hematoma volume and less severe stroke syndromes, but not a lower case-fatality when compared to VKA-ICH.^7^ Restoration of vitamin-K-dependent clotting factors in VKA-ICH and normalization of the INR can be achieved rapidly by treatment with four-factor prothrombin complex concentrate (PCC), which has shown to be superior to fresh frozen plasma.^8^ Rapidly achieving normalization of the INR appears to be associated with a lower risk of hematoma expansion,^9^ so the mantra of “time is brain” applies to ICH as well. Clinicians should thus work to minimize delays in care processes, and the use of point-of-care INR testing, agreed protocols, and rapid access to PCC has all been shown to help.^10^ Ambulance pre-alerting of all cases of suspected stroke on anticoagulants can reduce door-to-scan time significantly.^11^ Dabigatran can be reversed with idarucizumab, a monoclonal antibody fragment that leads to a rapid and sustained reversal of the anticoagulant effect.^12^ Until recently, the anti-Xa agents (apixaban, edoxaban, rivaroxaban, and betrixaban) have not had a licensed and specific reversal agent, and PCC has been recommended in guidelines.^13,14^ Healthy volunteer studies have shown that PCC at a dose of 50 IU/kg can correct endogenous thrombin potential and may reduce bleeding time.^15^ Observational studies without comparator arms have reported experience in giving PCC at lower doses in ICH and report effective hemostasis in around two-thirds and thromboembolic complications in 2.4–7.6% of patients.^16,17^ Andexanet alfa is a modified recombinant inactive form of human factor Xa, which binds specifically to anti-Xa agents and is licensed for use in life-threatening and uncontrolled bleeding in patients treated with apixaban and rivaroxaban.^18^ Seventy-one cases with relatively small ICH volume were included in a single-arm clinical trial of andexanet alfa and 23% had expansion >35% by 12 h and 10.9% of patients with ICH had a thrombotic event within 30 days of treatment.^18,19^ The lack of a comparator arm makes interpretation difficult but this will be addressed by the ongoing ANNEXa-I trial (andexanet alfa vs. standard care in ICH).

Prior antiplatelet use is associated with a larger baseline ICH volume, more hematoma growth, increased mortality but not a worse long-term functional outcome^20^ so may represent a therapeutic target. The PATCH trial randomized 190 patients with ICH taking antiplatelet drugs to platelet transfusion vs. standard care and showed, somewhat surprisingly, that platelet transfusion led to significantly higher death and dependence at 90 days so should thus be avoided for this indication.^21^ Although other treatments such as desmopressin are under investigation, aside from discontinuation of antiplatelets in the acute phase, there are currently no treatments of benefit in antiplatelet-associated ICH.

Although there is currently insufficient evidence to recommend routine use in ICH patients with normal clotting, recombinant activated factor VII (rFVIIa) and tranexamic acid may warrant further investigation. Tranexamic acid led to modest but significant reductions in early death, hematoma expansion and appeared safe in the large, phase III TICH-2 trial, but did not improve the modified Rankin Scale (mRS) at 90 days.^22^ Similarly, the FAST trial of rFVIIa showed a reduction in hematoma expansion but no improvement in 90-day mRS as well as an increase in thromboembolic complications at the highest dose.^23^ Further trials are either underway or planned, and targeting subgroups with the highest risk of hematoma expansion (e.g. very early after symptom onset) may lead to improvements in long-term clinical outcomes.

## Blood pressure management

High blood pressure (BP) occurs in 75% of patients with acute ICH, where impaired cerebral autoregulation and activation of the autonomic nervous system contribute to the hypertensive response.^24^ Persistent or episodic high BP may contribute to ongoing bleeding, and observational studies support there being an association with adverse outcomes, including growth of the underlying hematoma, and subsequent death and disability.^25^

Several randomized controlled trials have tested various targeted BP-lowering strategies in acute ICH patients.^26–30^ The first large trial to test intensive BP lowering in acute ICH, INTERACT2, reported a modest benefit from treatment on 90-day functional outcome and health-related quality of life,^28^ and international guidelines were updated to recommend BP lowering in selected patients.^13,31^ However, the second trial, ATACH-II, found no effect of treatment on 90-day death and major disability and that renal adverse events were more common in the treatment group.^30^

Meta-analyses of study-level data from these and other small trials reported no benefit of treatment on death and major disability, despite modest reductions in hematoma growth.^32,33^ However, these analyses did not account for the manner in which BP was lowered: although INTERACT2 and ATACH-II had similar designs (Table 1), BP was probably lowered more aggressively in ATACH-II, raising questions about the optimal approach to balance potential benefits (reductions in hematoma growth, improved function) and harms (cerebral, cardiac, or renal ischemia) from intensive BP lowering.

**Table 1.** Comparison of two large trials that tested intensive vs. guideline blood pressure lowering in acute intracerebral hemorrhage

	INTERACT2	ATACH-II
Eligibility		
Time window	<6 h	<4.5 h
Baseline SBP	150–220 mm Hg	180–240 mm Hg
Notable exclusions	Poor prognosis (death expected within 24 h)	Those with IVH where blood completely fills one lateral ventricle; recent warfarin use
Intervention		
Target	Lower mean SBP to <140 mm Hg within 1 h of randomization and maintain for seven days	Lower minimum SBP to 120–139 mm Hg within 2 h of randomization and maintain for 24 h
Agent	Physician discretion (locally agreed protocol)	IV nicardipine; rescue agents allowed
Treatment cessation	Stop IV treatment is SBP < 130 mm Hg	Stop treatment if SBP < 110 mm Hg
Control group	Target mean SBP < 180 mm Hg	Target minimum SBP 140–179 mm Hg
Findings	No effect of treatment of death or dependency (mRS scores 3–6); modest benefit of treatment on function (ordinal mRS scores) and health-related quality of life	No effect of treatment on death or major disability (mRS 4–6); preponderance of renal adverse events in treatment group

SBP: systolic blood pressure; IV: intravenous; IVH: intraventricular hemorrhage; mRS: modified Rankin Scale, where 0 = no disability and 6 = death).

**Box 1.** Reversal of anticoagulants

**Why?** Anticoagulants are associated with more hematoma expansion and worse outcomes. DOACs are increasingly prescribed and require different reversal strategies.** Which agent?** For VKA-ICH, PCC rapidly restores normal coagulation. Dabigatran should be reversed with idarucizumab. Andexanet alfa is licensed for life-threatening bleeding with rivaroxaban and apixaban. PCC is an alternative treatment for ICH on anti-Xa agents.** When?** Rapid correction of clotting is associated with a lower risk of hematoma expansion, so “time is brain.”** How?** Ambulance pre-alert for suspected stroke on anticoagulants, point-of-care testing, immediate access to reversal agents, and locally agreed protocols can speed reversal.

**Box 2.** Blood pressure lowering

**Why?** BP lowering may reduce hematoma growth and improve clinical outcomes: a large randomised controlled trial, INTERACT2, reported modest benefits of early (within 6 h of onset), targeted (systolic BP < 140 mm Hg) BP lowering in mild-to-moderate severity acute ICH.** How much?** During the first 24 h of treatment, careful (avoiding reductions ≥60 mm Hg within 1 h), targeted (as low as 120–130 mm Hg), and sustained (minimizing variability) BP lowering may be optimal.** When?** Ultra-early (<2 h of symptom onset) intensive BP lowering may have the most potential for reducing hematoma growth; however, uncertainties remain about the safety of pre-hospital BP lowering in acute ICH.** Which agent?** There is little evidence on which to base decisions about the preferred choice of agent. Locally agreed protocols tailored to clinicians’ familiarity and preferences are reasonable.

**Box 3.** Neurosurgery

**Why?** Surgical evacuation of supratentorial ICH may improve clinical outcomes, provided successful volume reduction can be achieved: a large randomized controlled trial, MISTIE 3 reported no improvement in functional outcomes although there was mortality benefit. Patients achieving the surgical goal of ≤15 mL residual ICH volume had improved functional outcome.** Who?** Hematoma evacuation may be more effective in certain subgroups, specifically patients in the moderate GCS range (5–12), with larger hemorrhage volumes (>30 mL). External ventricular drainage is advised in patients with IVH-associated hydrocephalus and decreased level of consciousness. Evacuation of cerebellar hemorrhage with neurologic deterioration or brainstem compression and/or hydrocephalus is recommended.** When?** Hematoma evacuation and in particular MIS may be more effective when performed between 7 and 24 h after onset although times to evacuation up to 72 h demonstrate efficacy over conventional therapy.** Which procedure?** Meta-analyses of studies evaluating surgical treatment report even higher likelihood of good functional outcome and lower risk of death with MIS (vs. craniotomy) compared to medical treatment only. There is little evidence about the preferred surgical procedure.

In analyses of pooled patient-level data from INTERACT2 and ATACH-II, linear associations existed for mean and variability of systolic BP during the first 24 h of treatment, whereby lower (as low as 120–130 mm Hg) and stable levels of systolic BP were safe and associated with favorable functional outcome.^34^ However, in secondary analyses, inverted U-shaped curves were apparent between categories of magnitude of systolic BP reduction within the first hour and death and neurological deterioration, suggesting harms may exist where large reductions (≥60 mm Hg) occurred within the first hour of treatment. Therefore, careful, targeted and sustained BP reductions may be beneficial, but reverse causality cannot be excluded. Furthermore, included patients had mild-to-moderate severity ICH, so caution should be applied in patients with large hematomas and severe neurological impairment.

Regarding timing of the treatment, a recent post-hoc analysis of ATACH-II showed that a subgroup of patients who received ultra-early (time from symptom onset to treatment <2 h) intensive BP lowering had less hematoma growth and better functional outcomes.^35^ However, subgroup analyses of the RIGHT2 trial that tested ultra-early (median time from symptom onset to randomization 74 min) use of topical nitrates in acute stroke in the pre-hospital setting showed a non-significant trend toward harm in ICH.^36^ These findings raise questions about the potential for ultra-early vasodilators to interrupt important hemostatic mechanisms^37^ and require further randomized evidence for confirmation.

There are no reliable data to support the choice of BP-lowering agent in acute ICH. In INTERACT2, alpha-adrenoreceptor blockers were the most frequently used agent in the treatment group; however, nearly half of all participants were treated with multiple agents. Data from several randomized trials, where alpha- and beta-adrenoreceptor blockers, angiotensin receptor blockers, calcium channel blockers, magnesium, and nitrates were used are available, and the results of a patient-level meta-analysis are awaited.^38^ Until robust data are available, locally agreed protocols that take into account clinicians’ familiarity and preferences are reasonable.

## Neurosurgery

**Figure 1.**
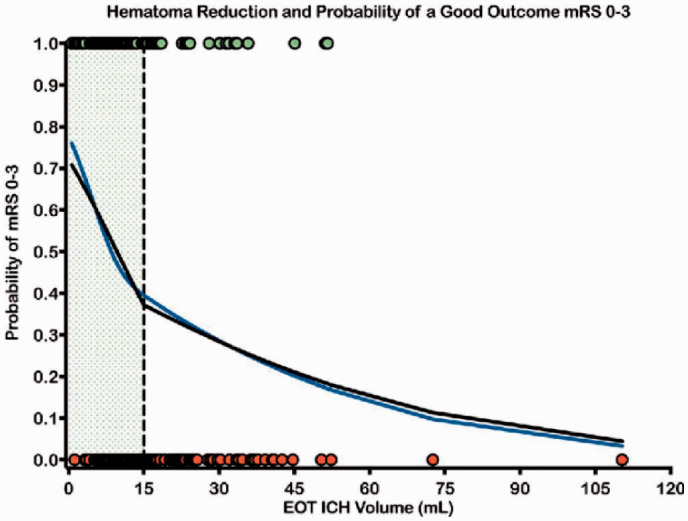
Cubic spline (blue line) and linear spline (black line) regression analyses showing the relationship of hematoma reduction (EOT ICH Volume) to the probability of having a good outcome (green dots), mRS 0–3, (vs. a poor outcome – red dots) at one year. Clot volume reduction beyond the 15 mL goal increased the chances of improved functional outcome by 10% for each additional milliliter removed (*p* = 0.002). Reprinted from Awad et al., Surgical performance determines functional outcome benefit in the Minimally Invasive Surgery Plus Recombinant Tissue Plasminogen Activator for Intracerebral Hemorrhage Evacuation (MISTIE) procedure, *Neurosurgery* 2019; 84: 1157–1168, by permission of the Congress of Neurological Surgeons.

Surgical hematoma evacuation is hypothesized to correct parenchymal displacement, decrease intracranial pressure (ICP), and mitigate neurotoxic and inflammatory cascades, but evidence to date is inconclusive. The STICH trial compared early craniotomy and hematoma evacuation with initial conservative management in supratentorial ICH and showed no overall benefit in mortality or favorable outcome at six months.^39^ STICH II compared early surgery in a more selected population of lobar hemorrhage without intraventricular hemorrhage (IVH) hypothesized to be more likely to benefit, but surgery again showed no overall benefit.^40^ More recently, there has been considerable interest in minimally invasive surgery (MIS), and multiple techniques are available, including image-guided (stereotactic) aspiration of the hematoma with or without the use of thrombolytic drugs, craniopuncture, mechanical clot disruption, and endoscopic removal including with continuous focal ultrasound delivered directly into the ICH. Refinement of MIS procedures is beginning to challenge clinical equipoise regarding volume-reduction therapy for ICH even for deep hemorrhage. MISTIE III, the largest clinical trial of MIS to date, demonstrated an improvement in mortality, but not functional outcome at one year.^41^ A significant improvement in functional outcome was suggested by a post-hoc analysis of MISTIE III patients with an ICH volume ≤15 mL at the end of treatment (Figure 1),^42^ suggesting that achieving adequate hematoma clearance may be crucial in achieving improved outcomes.

Systematic reviews and meta-analyses of studies evaluating surgical treatment in supratentorial ICH have consistently concluded that the likelihood of survival and good functional outcome is significantly higher in patients undergoing any surgical intervention when compared to medical treatment only and even higher in patients undergoing MIS compared to medical treatment.^43-45^ These interventions may be more effective in certain subgroups, specifically patients in the moderate Glasgow Coma Scale (GCS) range (5–12) with hemorrhage volume > 30 mL and undergoing surgery between 7 and 24 h.^44^ Whist early surgery seems better, the potential for reducing secondary brain injury from hematoma volume, expansion, intracranial hypertension, and perihematomal edema must be balanced against with the potential risk of early post-surgical bleeding.

Decompressive hemicraniectomy without hematoma evacuation can relieve elevated ICP and midline shift caused by mass effect and associated PHE. Small case series, case–control studies, and a meta-analysis report improved survival with variable effects on functional outcomes.^46^ The SWITCH trial compares decompressive surgery to best medical treatment only in patients with supratentorial ICH.

Though there have been no randomized clinical trials of surgical evacuation in cerebellar ICH, it is recommended for patients with neurologic deterioration, brainstem compression, and/or hydrocephalus.^13^ This concept has been challenged by an individual patient data meta-analysis of four observational ICH studies with 578 patients with cerebellar ICH, reporting no association between surgical evacuation and improved functional outcome at three months on propensity score-matched analysis.^47^ Survival, however, was improved, with greater benefit for cerebellar hematoma volume >15 mL.

IVH occurs in 40–45% of ICH and is associated with worse functional outcomes.^48^ External ventricular drainage is recommended in cases of obstructive hydrocephalus, which complicates approximately half of IVH.^49^ CLEAR III, a large randomized clinical trial comparing intraventricular alteplase with intraventricular saline for obstructive IVH, found no safety concerns, but no significant benefit was found on the primary outcome of mRS 0–3 at 180 days between treatment groups.^50^ Similar to ICH, extent of IVH reduction appears to be a critical factor; significant improvement in functional outcomes occurred only in the subgroup with >85% IVH clot removal.^50^

## Supportive management and rationale for bundles of care

In addition to the specific treatments above, the level of supportive care ICH patients receive in the acute phase may have a considerable impact on survival and recovery. Difficult decisions may need to be made quickly regarding appropriate levels of supportive care, ranging from admission to critical care with full organ support, to care on a stroke or neurosurgical unit, to withdrawal of active care and palliation. Such decisions depend on our ability to reliably predict prognosis, and an overly pessimistic view may lead to inappropriate limitations of care—a self-fulfilling prophecy. Evidence suggests that clinicians are more likely to commence early palliation for ICH compared to ischemic stroke, after taking demographics, premorbid health, and disease severity into account.^51^ Do-not-resuscitate (DNR) orders are widely used in ICH patients and have been shown to be independently associated with a worse outcome,^52^ with evidence that they may lead to limitations in care beyond their intended purpose.^52^ A multicenter prospective observational study has tested a policy of avoiding DNR orders in the first five days of care in a subgroup of patients with severe ICH (GCS ≤ 12).^53^ The observed 30-day case fatality of 20.2% was far lower than the 50% predicted by the ICH score, and by 90 days, mRS 0–3 was achieved by 29.9%.^53^ Multiple grading scales for ICH have been reported and are moderately predictive of survival and functional recovery,^54^ but there is some evidence that clinician judgement may be better.^55^ Given the difficulty in accurately predicting outcome and the effects of DNR orders, US guidelines recommend active supportive care and the postponement of DNR orders until the second full day of hospitalization.^13^

There is clear evidence that patients with ICH benefit at least as much as ischemic strokes from care on a dedicated stroke unit.^56^ For more unwell patients who may require multi-organ support, admission to a critical care unit should be considered, and observational studies suggest that care on a dedicated neurological critical care unit is associated with a lower mortality than care on a general critical care unit.^57^

**Figure 2.**
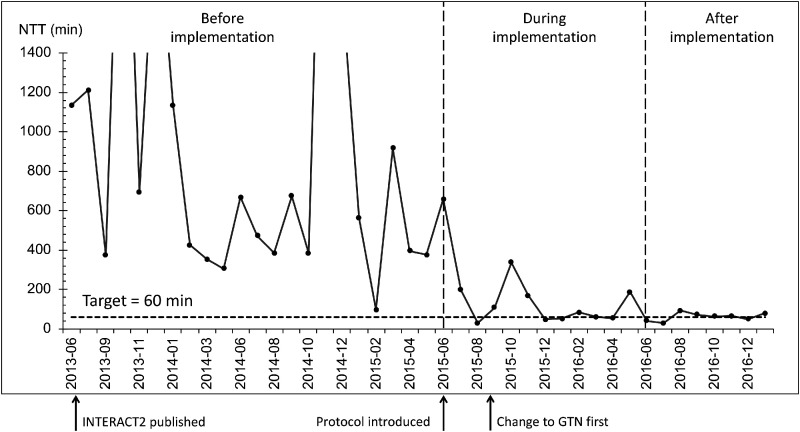
Run chart demonstrating progress in reducing the needle-to-target (NTT) time for intensive BP lowering in ICH before, during and after implementation of the ABC care bundle at Salford Royal Hospital, UK. The process target of 60 min is shown by a dashed horizontal line. Each point is the mean NTT for the month when BP lowering was attempted with parenteral medication. Publication of INTERACT2 did not alter management until a standardized protocol was introduced at bundle implementation. Further gains were achieved on switching to glyceryl trinitrate as the first-line drug.

It is well recognized that effective and widespread implementation of evidence-based care is challenging, ultimately limiting the benefit of a proven intervention in day-to-day clinical care.^58^ This “translational gap” can be especially hard to bridge where interventions are complex and challenging to implement such as the time-pressured, hyperacute management of ICH. Concerted and focused efforts may be required to improve care processes, with rapid and continuous collection and monitoring of process data, testing of changes to overcome contextual and disease-specific barriers, and ultimately working toward pre-defined process targets. An example of this approach is the implementation of the “ABC” care bundle at a regional comprehensive stroke center in the UK.^5^ The ABC bundle (standing for Anticoagulant reversal, BP lowering, Care pathway for referral to neurosurgery) served as a means of focusing the stroke team on delivering these key interventions rapidly and consistently. Clear-process targets were designed for each component, and rapid data collection was established and changes introduced to improve (Figure 2). This led to a marked reduction in 30-day case fatality (44% reduction after implementation vs. before) that was not accounted for by secular trends or case mix.^5^ Despite not being part of the ABC bundle, a reduction in DNR orders and improved access to critical care mediated the observed improvement in survival. An ICH care bundle focused on physiological control is currently being tested in the INTERACT3 cluster-randomized stepped-wedge trial in China with plans to expand to additional countries soon. As further evidence emerges for single interventions in ICH, similar approaches may be needed to accelerate and maximize effective implementation, with care bundles being adapted to incorporate new evidence.

## Conclusion

Despite the relatively poor prognosis of ICH, anticoagulant reversal, BP lowering, surgery in carefully selected cases and specialized supportive care may improve outcomes. These interventions can be challenging to deliver optimally, and a care bundle approach with proactive review of process data and improvements to care processes may have a significant impact on outcome. As promising new treatments emerge, they should be incorporated into care bundles to ensure rapid and effective implementation.

## Summary boxes
